# Pharmacological Targeting of Pore-Forming Toxins as Adjunctive Therapy for Invasive Bacterial Infection

**DOI:** 10.3390/toxins10120542

**Published:** 2018-12-17

**Authors:** Tamara Escajadillo, Victor Nizet

**Affiliations:** 1 Biomedical Sciences Graduate Program and Department of Pediatrics, School of Medicine, UC San Diego, La Jolla, CA 92093, USA; tescajadillo@ucsd.edu; 2 Department of Pediatrics and Skaggs School of Pharmacy and Pharmaceutical Sciences, UC San Diego, La Jolla, CA 92093, USA

**Keywords:** Pore-forming toxin, bacterial infection, virulence factor, pharmacology, adjunctive therapy

## Abstract

For many of the most important human bacterial infections, invasive disease severity is fueled by the cell damaging and pro-inflammatory effects of secreted pore-forming toxins (PFTs). Isogenic PFT-knockout mutants, e.g., *Staphylococcus aureus* lacking α-toxin or *Streptococcus pneumoniae* deficient in pneumolysin, show attenuation in animal infection models. This knowledge has inspired multi-model investigations of strategies to neutralize PFTs or counteract their toxicity as a novel pharmacological approach to ameliorate disease pathogenesis in clinical disease. Promising examples of small molecule, antibody or nanotherapeutic drug candidates that directly bind and neutralize PFTs, block their oligomerization or membrane receptor interactions, plug establishment membrane pores, or boost host cell resiliency to withstand PFT action have emerged. The present review highlights these new concepts, with a special focus on β-PFTs produced by leading invasive human Gram-positive bacterial pathogens. Such anti-virulence therapies could be applied as an adjunctive therapy to antibiotic-sensitive and -resistant strains alike, and further could be free of deleterious effects that deplete the normal microflora.

## 1. Introduction

The rapid emergence of antimicrobial resistant strains of bacteria has exceeded the rate at which anti-bacterial therapies are currently being produced. While the classical approach to drug development has focused on the enhancement of bactericidal properties of antibiotics, over-prescription coupled with non-adherence to treatment regimens has further contributed to the development of widespread resistance [1,2,3], underscoring the overarching need for the development of novel methods for combating bacterial infections. One possible approach that has gained attention over the past few years involves selecting bacterial virulence mechanisms as targets for therapy.

All bacteria rely on specialized virulence factors with which they can cause damage to host cells. While there exists a plethora of virulence mechanisms utilized by pathogenic bacteria to cause disease, the majority produce toxins that induce damage to either gain access to host cells for further proliferation, derive nutrients from host cells, or disrupt host cell immune function to increase their own survival, all of which may ultimately lead to host cell death. Pore forming toxins (PFTs) comprise about 25% of all known bacterial toxins, making them one of the largest classes of bacterial virulence factors [4,5,6]. All PFTs require binding in some way to a receptor on the host cell plasma membrane, where they oligomerize, form pores and alter membrane integrity [4,7,8]. While the mechanism of PFT pore formation may seem deceptively simple, PFTs can affect intracellular signaling cascades, dependent in part on the membrane structure the are bound to, and therefore can produce a variety of downstream responses, enhancing the pathogenicity of the bacteria that secrete them [5,6,8,9,10,11,12].

By preserving cell membrane integrity and viability of all host cells, including immune cells, PFT virulence factor neutralization could aid in facilitating pathogen elimination by normal immunity, while preserving the beneficial host microbiome. Given that anti-virulence strategies do not focus on directly killing the pathogen, they could provide the additional benefit of exerting less selective pressure, which may in turn result in a decreased need by the bacteria to develop resistance [13,14,15].

This review will focus on the non-classical approaches that have been developed toward reducing bacterial-induced damage to host cells through inhibition of secreted PFTs. These concepts have the potential to become important pharmacological strategies for improving treatment outcomes, used either independently or as an adjunct to classical antibiotic regimens.

## 2. Classification of Pore-Forming Toxins

PFTs can generally be classified into two large groups based on the secondary structure used to traverse the host cell plasma membrane, and are designated α-PFTs, for the creation of α-helixes, and β-PFTs, for the creation of β-barrels [16]. The α-PFTs use clusters of amphipathic and hydrophobic helices to form pores in the target membrane [17,18], and the archetypal members of this class are the colicins produced by *Escherichia coli* [19,20,21]. Additional α-PFT members include Cry toxins of *Bacillus thuringiensis* [22], diphtheria toxin of Corynebacterium diphtheriae [23], and exotoxin A produced by *Pseudomonas aeruginosa* [24].

β-PFTs represent the majority of currently identified bacterial PFTs and are more extensively studied due to the high stability of their inter-strand hydrogens, which allows the establishment of more precise classifications [25,26,27]. A subset of β-PFTs can be further classified into three separate groups: hemolysins, aerolysins, and cholesterol-dependent cytolysins (CDCs) [6]. CDCs require the presence of cholesterol during at least one step of their activity; they may also be referred to as thiol-activated cytolysins due to their reported sensitivity to oxygen [28]. The CDC family is particular large, with over 20 PFTs that share common structural motifs secreted by a range of Gram-positive and Gram-negative bacteria. Among the most widely studied are streptolysin O (SLO) from *Streptococcus pyogenes* [29], pneumolysin (PLY) from *Streptococcus pneumoniae* [30], intermedilysin (ILY) from *Streptococcus intermedius* [31], listeriolysin O (LLO) from *Listeria monocytogenes* [32,33], anthrolysin O (ALO) from *Bacillus anthracis* [34] and perfingolysin O (PFO) from *Clostridium perfringens* [35]. Commonly studied non-CDC β-PFTs include aerolysin from *Aeromonas hydrophila* [36] and α-hemolysin (Hla) from *Staphylococcus aureus* [37].

When their expression is activated to support key steps in bacterial pathogenesis, β-PFT monomers are secreted by their corresponding species. The β-PFT then binds to a receptor on the plasma membrane of the host cell, where it oligomerizes to form a pre-pore complex. This complex converts to the inserted pore complex upon insertion of the transmembrane β-sheet. The precise mechanistic details of pore formation have previously been reviewed in detail [5,17,25,27,28,38,39,40]. For the purposes of this review, we will primarily focus on inhibition and neutralization of β-PFTs secreted by important Gram-positive bacterial pathogens of humans.

## 3. Overview of Pharmacological Approaches to Pore-Forming Toxin Inhibition

**Figure 1:**
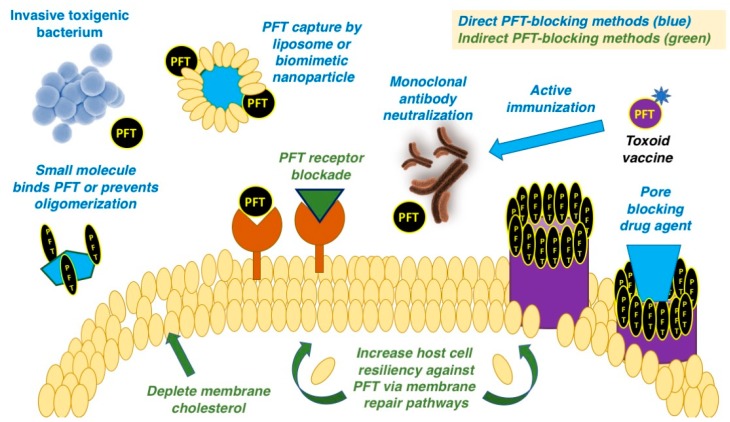
Overview of pharmacological strategies to counteract bacterial pore-forming toxin (PFT) virulence factors discussed in the present review. Promising examples of small molecule, antibody (passive or active immunization) and nanotherapeutic drug candidates that directly bind and neutralize PFTs, block their oligomerization or membrane receptor interactions, plug establishment membrane pores, or boost host cell resiliency to withstand PFT action have emerged.

Given the multi-step process of host cell engagement and pore formation after toxin secretion by the bacterium, there exist multiple stages at which a PFT toxin can be targeted for inhibition, both directly and indirectly. While direct toxin inhibition involves physical binding to the monomer to preclude interaction with the host cell or interruption of the oligomerization process, indirect methods have been described including prevention of binding to the host cell receptor, pore blockade or stimulating membrane repair pathways to boost the cell’s ability to mitigate toxin-induced damage (Figure 1). Small molecules, some repurposed from other areas of medicine, liposome and nanoparticle platforms, and monoclonal antibodies or toxoid vaccine-induced humoral responses, comprise the repertoire of agents discussed in the ensuing sections.

## 4. Direct Binding or Sequestration of Pore-Forming Toxins

Direct binding to the secreted β-PFT monomer constitutes one of the most straightforward approaches to neutralization and has been one of the most extensively explored within the realm of bacterial infections. In addition, the process of pore formation is dynamic, with different structural and functional states existing in the path to pore formation [41]. Once the β-PFT has bound to its receptor on the host cell, it undergoes conformational changes and oligomerizes to form a lytic transmembrane pore domain and insert itself into the plasma membrane. The elucidation of these dynamic processes has benefited greatly from continual advances in electron microscopy, time-lapse atomic force microscopy, and X-ray crystallography, which together have allowed direct observation and precise characterization of these different stages [42,43,44,45,46,47].

### 4.1. Passive Antibody Neutralization of Pore-Forming Toxins

A variety of molecules have been discovered that are capable of directly binding to PFTs, most prominently monoclonal antibodies (mAbs). Despite being the largest category of PFT-neutralizing agents, mAbs have not been explored as extensively in the realm of antibacterial therapeutics as they have in other areas of medicine, such as oncology and viral infections [48]. mAbs are generated from an isolated B-cell clone expressing a single isotope, and then evaluated through various high-throughput protocols for epitope binning and relative affinity ranking to identify functional clones for hybridoma development [49,50,51].

Because of its medical importance and established role in the pathogenesis of pneumonia, skin necrosis and systemic infection, β-PFT Hla (α-toxin) of *S. aureus* has been a leading target of antibody therapeutic programs. Encouraging results from passive immunization studies with rabbit polyclonal Hla-specific antisera, generated using a purified inactive form of the toxin (HlaH35L) as the immunogen, established a first proof-of-principle [52,53], inspiring the development of several ant-Hla mAbs using large-scale libraries and high-throughput screening methods. For example, AR-301 (Salvecin™, Aridis Pharmaceuticals, Inc, San Jose, CA, USA) is a fully human monoclonal IgG1 antibody that was evaluated in a phase 2a clinical trial as adjunctive therapy to standard antibiotics for severe culture-confirmed *S. aureus* pneumonia in ICU patients. Post-hoc subgroup analysis suggested potential benefits in reducing the duration of ventilator support in intubated patients, a trend toward faster microbiological eradication, no serious adverse events attributed to the drug, and a plasma half-life of four weeks; however, larger randomized trials would be required to better define safety and efficacy [54]. The mAb MEDI4893 (MedImmune, LLC) binds Hla through an epitope in the “rim” domain to exert a dual mechanism of neutralization: (a) preventing the toxin from adopting a pore-forming heptameric transmembrane conformation required for host cell lysis, and (b) inhibiting Hla binding to its cellular receptor, A Disintegrin and metalloproteinase domain-containing protein 10 (ADAM10) [55,56,57]. The safety, tolerability, pharmacokinetics, anti-Hla-neutralizing activity of MEDI4893 have recently been explored in a phase 1, double-blind, dose escalation study [58].

Still in preclinical development, other mAbs against Hla include: (a) 2A3 and its affinity-optimized variant LC10, which reduce disease severity in a murine model of *S. aureus* pneumonia [59,60], (b) mAbs 7B8 and 1A9 generated against the nontoxic Hla_H35L_ mutant that prevent oligomerization of the β-PFT and provide a high degree of protection against *S. aureus* pneumonia [61], and (c) high-affinity mAb LTM14, derived from a phage display library, which blocks the lytic activity of Hla in a dose-dependent manner in vitro [55].

In addition to Hla, *S. aureus* strains can secrete an array of bicomponent β-PFTs, which are comprised of two subunits (S and F) with β-barrel structure that achieve pore-forming configuration after binding to specific cell receptors followed by hetero-oligomerization of the two subunits at the plasma membrane [62,63]. The oligomeric β-PFTs then create the plasma membrane pore leading to pathological ion fluxes and activation of cell death pathways. These bicomponent β-PFTs are often termed leukocidins, because of their capacity to target and functionally inactivate host neutrophils and monocyte/macrophages and include Panton-Valentine leukocidin (Luk-SF-PV or PVL) that has been epidemiologically linked to severe infections in epidemic clones of community-acquired *S. aureus* [62,63]. Indeed, passive transfer of rabbit immunoglobulin raised against LukS-PV alone protects against *S. aureus* sepsis [64]. In a search for targeted therapeutics, the mAb ASN-1 was isolated by screening a human IgG1 antibody library with a yeast selection system and found to bind four members of the *S. aureus* leukotoxin family of cytotoxins: LukSF-PV (Panton-Valentine leucocidin), LukED, HlgAB, and HlgCB, in addition to Hla [65]. A second mAb ASN-2 neutralized the additional *S. aureus* leukotoxins LukGH. ASN-1 and ASN-2 mAb were then combined in the formulation ASN-100 (Arsanis, Inc.) [66], with no dose limiting toxicity observed to date [67]. In other approaches, humanized heavy chain-only antibodies (HCAb) were generated against *S. aureus* leukocidins LukS-PV and LukF-PV and validated in vitro and in vivo, with the additional benefit of preventing toxin binding and pore formation for γ-hemolysin C (HlgC) [68].

Work to develop antibody neutralization strategies against β-PFTs from other key human pathogens has been more preliminary. The murine mAbs PLY 4 and PLY 7 effectively target the CDC PFT *S. pneumoniae* pneumolysin (PLY), and are directed against epitopes that have dual actions to reduce cytolytic activity and block binding to host cells, although they have not advanced to humanization protocols or clinical trials yet [69,70]. Passive administration of monoclonal antibodies to *B. anthracis* anthrolysin O (ALO) may provide a measure of host protection under specific circumstances. One mAb (64F8) reduced the cellular toxicity of rALO in vitro, and the combination of mAbs 64F8 and 80C9 was more effective than either mAb alone in prolonging survival in a murine systemic anthrax infection model [71]. Polyclonal antibodies raised in rabbits against mutated Y30A-Y196A *C. perfringens* Epsilon toxin (Etx), a β-PFT that causes enterotoxemia, neutralize the purified wild-type toxin in vitro [72], but have yet to proceed to mAb development or translational studies in live infection models.

### 4.2. Small Molecules that Bind or Inhibit Toxin Assembly

While there are several small molecule natural compounds reported to reduce the level of Hla production by *S. aureus* [73,74,75], a few have been studied for their direct toxin neutralizing activity, including baicalin. Baicalin is a flavonoid compound isolated from the traditional medicinal herb *Scutellaria baicalensisa* (Chinese skullcap), which binds Hla directly and inhibits its hemolytic activity by restraining the conformational change of the binding cavity [76]. Other flavonoids reported to inhibit Hla include apigenin, chrysin, kaempferol, luteolin, and quercetin, the natural compounds *trans*-resveratrol and betulinic acid [77], and the O-methylated flavone oroxylin A, which bind to the Hla active site (Thr11, Thr12, Ile14, Gly15 and Lys46) to inhibit self-assembly of the heptameric transmembrane pore [78]. Morin hydrate, another bioflavonoid, was predicted to bind to residues I107 and T109 and induce a conformational change that leads to inhibition of the self-assembly of the heptameric transmembrane pore [79]. In silico-based approaches based on key residues involved in the formation of the pore complex were used to design potential peptides for Hla inhibition, revealing the IYGSKANRQTDK peptide to both efficiently bind and disturb dimer formation [80].

Several natural product-derived compounds interfere with the oligomerization of PLY on cell membranes, although their actions have not yet been evaluated in the clinical setting. Amentoflavone (AMF; 4′,4‴,5,5″,7,7″-hexahydroxy-3‴,8-bi- flavone), is a flavonoid extracted from *Selaginella tamariscina* and other plants widely used in traditional Chinese medicine. AMF blocks the PLY oligomerization process and inhibits its cytolytic activity by binding at the cleft between PLY domains 3 and 4. [81]. Verbascoside is a phenylpropanoid glycoside that does not exhibit bacteriostatic activity, but as shown through molecular dynamics simulations and mutational analysis, inhibits PLY mediated cytotoxicity by a similar domain 3–4 cleft binding [82]. In vitro studies of the flavonoid apigenin showed direct interaction with PLY, dose-dependent attenuation of hemolytic activity, and significant protection in a murine model of pneumococcal pneumonia [83]. Finally, β-sitosterol, a plant-derived cholesterol mimic, binds PLY with high affinity via Thr-459 and Leu-460, without intervening in oligomerization [84].

Allicin, the most abundant thiosulfinate molecule found in garlic extract, blocks SLO hemolytic activity, likely by binding the cysteine residue in the toxin’s binding site [85]. Glycan array analysis determined that SLO significant bound to 47 glycan structures, and upon flow cytometric analysis, free lacto-*N*-neotetraose (LNnT), which binds to SLO domain 4, blocked RBC binding, highlighting glycans as a source for other possible inhibitors of β-PFT function [86]. Via molecular modeling and mutational analysis, fisetin, a dietary flavonoid, directly engaged loops 2 and 3 of LLO, blocking cholesterol binding and reducing its oligomerization and hemolytic activity [87]. From molecular docking analysis of LLO using a library containing 200,000 drug-like compounds, one molecule (1-(4-Cyclopent-3-enyl-6, 7-dihydroxy-8-hydroxymethyl-nona-2, 8-dienylideneamino)-penta-1,4-dien-3-one) inhibited LLO oligomerization, with strong predicted binding properties and no undesirable toxicity [88].

### 4.3. Inhibition of Pore-Forming Toxins through Decoy Capture

A characteristic that all β-PFTs share in common is the requirement to bind the plasma membrane of the target host cell in some capacity. This principle inspired the development of liposomal targets that can serve as decoys for toxins by mimicking the lipid composition of natural host membranes. Liposomes are synthetic, spherical, nanoscale multilamellar or unilamellar bilayer vesicles composed of a variety of lipids that have been used for various commercial applications, including enhancing drug delivery and enhancing signaling for medical diagnostics [89,90]. These lipid layers can be modeled after the distinct microdomains known as lipid rafts which, while unstable in vivo, can be stably artificially created within liposomes. The liposomes can be optimized to enhanced selectivity for bacterial toxins by using specific concentrations of cholesterol:sphingolyelin (Ch:Sm) [91]. Liposomes (66 mol/% cholesterol) are able to bind CDCs and Hla, while a mixture of cholesterol:sphingolyelin liposomes (66 mol/% cholesterol) with sphingomyelin only liposomes (Ch:Sm + Sm) sequestered a larger array of bacterial toxins. One can see improved therapeutic efficacy of antibiotics used in combination with their liposomal formulations as adjunctive therapy during in vivo *S. aureus* and *S. pneumoniae* bacteremia models [91]. These studies served as the basis for the development of CAL02, a new liposomal formulation marketed by the Swiss-based company Combioxin SA. CAL02 recently completed Phase I clinical trials for neutralization of a large panel of bacterial toxins via recognition of the artificially engineered lipid rafts on the liposomes.

Despite the promising nature of liposomal toxin decoy capture platforms, these artificial nanoparticle formulations face many challenges, including rapid opsonization and clearance by macrophages and the need to tailor liposomal formulations to specific concentration ranges [92,93]. Various attempts to circumvent this problem by applying stealth coatings have raised the issue of off-target immunological responses [94]. These considerations recently led to the development of natural host cell membrane camouflaged nanoparticles, which significantly enhanced bioavailability. This characteristic is key for absorbing toxins in the bloodstream while providing the same complex surface chemistry of the biological cell from which they are derived [95]. RBC membrane-derived nanoparticles internally stabilized with a poly(lactic-co-glycolic-acid) (PLGA) core have been shown to bind and retain a variety of β-PFTs including Hla SLO, and LLO [96], and significantly reduced the ability of these toxins to induce host cell damage both in vitro and in mouse challenge models [96,97,98,99]. Conceivably, an expansion of this approach with the corresponding host target membranes has the potential to detoxify all membrane-damaging bacterial PFTs.

## 5. Inhibition of Host Cell Receptors or Uptake of Pore-Forming Toxins

PFTs recognize target cells by binding to different receptors including sugars, lipids and proteins on the plasma membrane, yet not all receptors have been identified, leaving this an ongoing area of research [6]. Research into the mechanism of action of *S. aureus* Hla has shown that the protein receptor ADAM10, a zinc-dependent metalloprotease, is required for toxin binding to the eukaryotic cell membrane and consequent toxicity [100]. The hydroxamate inhibitor GI254023X inhibits ADAM10 by fitting into the S1 specificity pocket [101], attenuating epithelial barrier dysfunction by preventing the cleavage of the junctional protein E-cadherin [102]. GI254023X treatment significantly decreased lesion size in a murine model of *S. aureus* necrotizing skin infection [103]. Co-receptors for another *S. aureus* β-PFT, leukocidin LukED, include the human immunodeficiency virus (HIV) co-receptor or CC-chemokine receptor type 5 (CCR5) and CXC-chemokine receptor type 1 and 2 (CXCR1 and CXCR2). Activity of LukED can be efficiently blocked using CCR5 receptor agonists and the FDA-approved HIV drug maraviroc [104,105].

Intermedilysin (ILY), a CDC from *Streptococcus intermedius*, in addition to cholesterol binds human complement regulator CD59, a glycosylphosphatidylinositol (GPI)-anchored cell-surface receptor, thereby promoting oligomerization and pore formation on the host cell membrane [11,106,107]. The crystal structure of CD59 bound to ILY was solved and guided synthesis of a peptide based on the binding site comprised of ILY residues 438-452; this peptide successfully competed for CD59 binding and inhibited ILY pore formation in vitro [108]. Another study characterized the mechanism of the 114-amino acid recombinant form of the 4th domain of intermedilysin (rILYd4) as another CD59 inhibitor. Upon binding to rILYd4, CD59 is internalized and is degraded in lysosomes within minutes, while the remaining rILYd4·CD59 complexes are shed from the cell [109]. Major binding targets for the *Aeromonas* β-PFT aerolysin are also GPI-anchored surface receptors, including T-lymphocyte protein (Thy-1) [12]. Given the ability of synthetic analogs of GPIs to inhibit binding of a related toxin (CAMP factor), this approach could conceivably apply to aerolysin as well [110]. LeX/sLeX glycans have recently been identified as receptors for the pneumococcal β-PFT PLY that contribute to RBC lysis. When cells were preincubated with anti-sLeX and anti-LeX monoclonal antibodies prior to PLY exposure, hemolytic activity was inhibited [86].

Cholesterol is widely understood to be essential for pore formation of CDCs and appears to play multiple roles, including targeting, promotion of oligomerization, triggering a membrane insertion competent form, and stabilizing the membrane pore [35]. It has been suggested that the extent of toxin binding and size of the oligomers is dependent upon the cholesterol concentration in the membrane [111]. Thus, it is possible that pharmacological approaches to reduce cell membrane cholesterol could have a beneficial effect of reducing β-PFT pore formation, especially with the CDC subclass of toxins. Statins, competitive inhibitors of 3-hydroxy 3-methylglutaryl coenzyme A (HMG-CoA), the rate-limiting enzyme in cholesterol biosynthesis, are mainstays of hyperlipidemia therapy in humans with risk factors for atherosclerosis [112]. Studies in a mouse model of sickle cell disease (SCD) suggested that one such drug, Simvastatin, protected host cells from the cytotoxic effects of PLY and other CDCs, including SLO and tetanolysin, a finding suggested to reflect the requirement of cholesterol for efficient pore formation and cytotoxicity [113]. Human airway epithelial cells treated with physiological serum concentration ranges of simvastatin in vitro also confirmed protection against PLY and Hla cytotoxicity, but this finding was not shared in other cell lines tested [114]. Since statins are known to have other pleiotrophic and immunomodulatory effects on cells, further experimentation with these drugs and other cholesterol-modifying agents in the context of β-PFTs is warranted. In this way, a mechanistic framework can be elucidated and inform potential therapeutic avenues.

## 6. Blockade of Pore Formation

Once the β-PFT has oligomerized and been inserted into the host cell membrane, the pores that are formed have a characteristic size and shape depending on the particular toxin inducing the damage [115]. The pore becomes a permeable channel and pathway for the flux of ions and other charged or polar molecules to cross the plasma membrane, which can alter host downstream signaling cascades at sub-cytolytic levels, or at higher doses provoke complete cell lysis [5,6,7,116,117,118]. Considering this deceptively simple shared outcome of β-PFT action, i.e., the creation of a membrane hole, a potential approach to counteract this insult arises in the direct physical obstruction or “plugging” of the hole [119,120].

One method that has been explored to block oligomeric pores is the use of cyclodextrins, cyclic oligomers of glucose that form water-soluble inclusion complexes with small and large molecules in a variety of biotechnology applications [121]. Selectively blockade of *S. aureus* Hla using β-cyclodextrin derivatives demonstrated that symmetry, size of the inhibitor and pore size are all important factors in determining efficacy [122,123]. For example, only β-cyclodextrin derivatives, and not α- or γ-derivatives, effectively inhibited Hla by mirroring the symmetry of the heptameric toxin. In other studies, the β-cyclodextrin derivative IB201 (ANBOβCD) blocked Hla channels irreversibly [123], and were reported to block the assembled Hla pore and decrease mortality in a murine model of *S. aureus* pneumonia [124]. A slightly different approach showed that two different salts of an isatin-Schiff base copper(II) complex, Cu(isapn), with perclorate—[Cu(isapn)](ClO_4_)_2_—or sulfonate—[Cu(isapn)](SO_4_)_2_, had significant anti-Hla activity by interacting with the constriction region of the pore and blocking it in an electrical potential-dependent manner [125]. Finally, a high-throughput screening of a 151,616-compound library identified three compounds, N-cycloalkylbenzamide, furo[2,3-b]quinoline, and 6H-anthra[1,9-cd]isoxazol, that appeared to inhibit *C. perfringens* Etx toxin by blocking the active pore, since cytotoxicity was reduced without direct toxin binding or interference with the toxin oligomerization process [126].

## 7. Increasing Host Cell Resiliency Against Pore-Forming Toxin Action

After attack by a PFT, the host cell can respond to this insult through several mechanisms that depend upon the type and concentration of the PFT, as well as the nature of the host cell(s) affected, since both factors differentially influence downstream stress response pathways and signaling events [8]. Although high concentrations of PFTs may cause wholescale host cell lysis, at lower toxin concentrations, survival of intoxicated cells is well documented and reviewed [6,8,127]. Understanding the specific mechanisms by which the host cell resists PFT damage can reveal new targets for pharmacological intervention.

Resealing of the membrane pore through regulation of membrane lipids, control of cytoskeletal dynamics, enhancement of blebbing and microvesicle shedding are some of the attractive options for further investigation [128,129,130,131]. For example, the decrease in cytoplasmic potassium after pore formation promotes inflammasome activation through caspase-1, leading to maturation of the important pro-immune and pro-inflammatory cytokine IL-1β to combat infection. This same potassium efflux influences lipid membrane biogenesis gene regulator sterol regulatory element binding protein 1 (SREBP1), a key orchestrator of membrane repair processes [132]. Another study reported that the pretreatment of lung epithelial cells with interferon-alpha (IFN-α) prior to challenge with *S. aureus* Hla prevents cell-death by modifying lipid metabolism and increasing protein synthesis and fatty acid activity, noting that these changes were independent of caspase-1 or mitogen-activated protein kinases [133].

A key virulence attribute of PLY is its ability to impair pulmonary barrier function by disrupting epithelial tight junction integrity to increase alveolar permeability [134]. A recent study revealed two peptides derived from host mediators that may counteract these negative effects of PLY. First, growth hormone-releasing hormone (GHRH) agonist JI-34 was found to enhance epithelial sodium channel (ENaC) function and capillary resistance in a cAMP-dependent manner [135]. Secondly, the TNFα-derived TIP peptide AP301, currently in phase 2a clinical trials, blunted activation of the enzymes protein kinase C-α and arginase 1, which induce hyperpermeability of the capillary endothelium following PLY exposure, both in vitro and in an in vivo mouse model [135].

A screen in the roundworm *Caenorhabditis elegans* for mutants resistant to the PFT crystal protein (Cry) revealed that induction of hypoxia-inducible factor-1 (HIF-1), a transcription factor expressed by all metazoan species and master regulator of oxygen homeostasis [136], can protect cells against cytotoxicity [137]. HIF induces the transcription of genes involved in the innate immune response such as IL-1β, IL-8, TNFα, iNOS and the antimicrobial peptide cathelicidin LL-37, which could explain the enhanced survival after PFT intoxication [138]. Under normoxic conditions, HIF is hydroxylated by prolyl hydroxylases (PHDs) and consequently degraded by a ubiquitin-ligase complex in the host cell proteosome [139]. AKB-4924 (Akebia Therapeutics), a PHD inhibitor that stabilizes HIF-1 levels and increases cutaneous innate defense against *S. aureus* infections [140], and it is conceivable that protection against Hla, a major virulence factor during skin infection, contributes significantly to the drug’s pharmacological efficacy.

Recently, high-throughput genetic screens in human cells have been deployed to discover novel host factors required for bacterial PFT toxicity [141,142]. Insertional mutagenesis screens in human haploid cells coupled to validation by CRISPR-Cas9 gene deletion studies revealed plekstrin-homology domain containing protein 7 (PLEKHA7) to be a novel mediator of Hla cytotoxicity [143]. Another gene-trap mutagenesis and RNA interference study concluded that Etx was capable of binding to the hepatitis A virus cellular receptor 1 (HAVCR1), providing new insights into the process of toxin-induced cell death [144] Finally, a host cellular defense mechanism suggested as a potential therapeutic target to protect against PFT injury is autophagy, a lysosomal process involved in maintaining cellular homeostasis through turnover of damaged or redundant proteins and organelles [145], which in its extreme form can result in autophagic cell death [146]. Numerous bacterial pathogens interfere with the autophagy process, but the interaction between autophagy and bacteria often depends on the type of bacteria and the type of PFT they secrete [146,147]. A review of selected FDA-approved drugs and pharmacological agents that modulate autophagy and could help improve the outcome of antibiotic treatment can be found here [148].

## 8. Inactivated PFT (Toxoid) Vaccines for Active Immunization

Knowledge derived from decades of studying PFT structure and pore architecture by x-ray crystallography and other structural biochemistry methods has informed the design of inactivated (toxoid) vaccines to prime the host immune system without inducing the deleterious effects of the PFT itself [6,149]. Pneumococcal polysaccharide conjugate vaccines, including the 7- and 13-valent forms of Prevnar and the 23-valent Pneumovax, have dramatically decreased the burden of severe disease in many populations [150]. However, development of broader protection among more than 90 serotypes of pneumococcus is desirable due to ongoing shifts in serotype epidemiology. For this reason, the highly conserved PLY has been regarded as an attractive protein antigen vaccine candidate [149,151,152]. Pneumolysin toxoid (dPly), formulated with another pneumococcal protein histidine-triad protein D (PhtD), provided protection against pneumococcal infection after active immunization in animal models [153,154,155]. Further phase I clinical trials in adults and toddlers showed that dPly + PhtD vaccine formulations were well tolerated and immunogenic when administered as standalone protein vaccines or combined with capsule polysaccharide conjugates [152,153,156]. Another promising PLY toxoid candidate is generated by deletion of alanine 146 and arginine 147 in the pore-forming region (ΔA146 PLY) [157], which does not produce an unwanted pro-inflammatory response in neutrophils [158]. Finally, a fusion protein created using L460D, a noncytolytic PLY toxoid incapable of binding cholesterol [159], when formulated together with conserved choline-binding protein A (CbpA), was broadly protective against pneumococcal infection, with the potential for additional defense against other meningeal pathogens expressing CbpA-like proteins [160].

In a murine model of *S. aureus* pneumonia, immunization with an Hla mutant possessing a single amino acid substitution (Hla_H35L_) led to decreasing burden and overall mortality upon challenge with the pathogen [52]. Similar protection following Hla_H35L_ vaccination was observed with reduced lesion size in a necrotizing skin infection model [53]. Reasoning that a single point mutation in Hla might not be considered ideally safe for use in a clinical setting, additional truncation mutant forms of the toxin have been evaluated as vaccine candidates using structure-based approach. Based on molecular modeling, a lead Hla vaccine candidate (AT-62aa) exhibited strong immunogenicity in mice when used with two clinically validated adjuvants (AlPO_4_ and GLA-SE) in models of *S. aureus* skin and soft tissue infections [161,162]. A chimeric bivalent vaccine using inactivated Hla and *S. aureus* iron surface determinant B (IsdB) has a stronger protective immune response than either protein alone [163]. Attenuated subunit vaccines using mutant forms of PFTs LukS-PV and LukF-PV subunits LukS-Mut9/LukF-Mut1 were highly immunogenic and demonstrate significant protection against *S. aureus* sepsis [64].

Certain noncytolytic LLO mutants (LLO W492A and LLO W491-492A) maintain binding capacity to the cell membranes with high affinity and are catabolized, processed, and presented efficiently by APCs to CD4+ or CD8+ T cells [164,165,166], although LLO is known to be strongly immunogenic, independent of its cytotoxicity [165], and its role has been explored more extensively in the context of live vaccine vectors [167]. Initial studies on *C. perfringens* Epsilon toxin (Etx) determined that the H149A mutation (Etx-H149A) could reduce, yet not abolish, toxicity [168], and inspired evaluation of the site-directed Etx mutant (Y30A-Y196A) as a potential recombinant vaccine antigen component. In vitro, Etx (Y30A-Y196A) significantly reduced cell binding and cytotoxic activities in MDCK.2 cells [72]. Studies on *S. pyogenes* SLO showed that a recombinant SLO derivate (rSLOmut) with mutated tryptophan residue in the membrane-binding loop W535A elicits protective immunity against lethal GAS challenge [169,170].

In a recent effort to maximize PFT vaccine potency and safety, alternative strategies employing non-denatured PFTs anchored to the aforementioned RBC membrane-coated nanoparticles have been explored. These “nanotoxoids” harboring Hla were capable of bestowing strong protective immunity in a murine lethal intoxication model with the native virulence factor [171,172]. The nanotoxoid vaccines were efficiently cleared with no additional toxicity after a period of two weeks and could potentially be used for a broad range of PFTs simultaneously in a multiplexed format, ultimately finding their way to the clinic using O-negative donor blood (or the patient’s own blood).

## 9. Conclusions

While the contribution of PFTs to the establishment and severity of many leading bacterial infectious disease has long been appreciated, significant steps to exploit this knowledge to improve patient outcomes is limited to proof-of-principle investigations with target cell lines, preclinical-stage in vivo studies in small animal models, and a few early clinical studies, predominantly in the realm of monoclonal anti-PFT antibodies. This review cites intriguing evidence that disarming bacterial pathogens through inhibition of their β-PFTs at various stages of secretion or host cell interaction (Table 1) could ameliorate disease pathology and constitute a viable adjunctive treatment option when classical antibiotics and supportive care fail to achieve rapid resolution. In and of themselves, PFT-neutralizing drugs would spare the normal microbiome from depletion associated with broad-spectrum antibiotic therapy, increasingly seen as an aggravating risk factor for many chronic inflammatory or autoimmune diseases. More detailed structure-function analysis of leading PFTs, coupled with a mechanistic understanding of the host cell stress response pathways they induce, can inform the optimal design and discovery of new agents in this class.

## Figures and Tables

**Table 1:** Potential therapeutic approaches to inactivate bacterial pore-forming toxins.

General Mechanism	Subclassification	PFT	Therapeutic Concept or Candidate	References
**Direct Binding and Inhibition**	Passive Immunization with Monoclonal Antibodies	*S. aureus* α-toxin (Hla)	mAbs MEDI4893, (MedImmune), LTM14. A3 and LC10, 7B8 and 1A9.	[52,53,54,55,56,57,58,59,60,61]
		LukS-PV, LukF-PV, γ-hemolysin C (HlgC)	AR-301 (Salvecin™) ASN-1 and ASN-2. ASN100 (Arsanis)	[62,63,64,65,66,67]
		*S. aureus* leukotoxins	Heavy chain-only antibodies	[68]
		Pneumolysin (PLY)	mAbs PLY 4 PLY 7	[69,70]
		Anthrolysin O (ALO)	mAbs 64F8 and 80C9	[71]
	Small Molecules that Bind or Inhibit Toxin Assembly	*S. aureus* α-toxin (Hla)	Baicalin, quercetin, trans-reservertrol, betulinic acid, orolyxins and other flavonoids, peptide IYGSKANRQTDK	[76,77,78,79,80]
		Pneumolysin (PLY)	β-sitosterol, apigenin, amentoflavone, verbascoside	[81,82,83,84]
		Streptolysin O (SLO)	Allicin, lacto-*N*-neotetraose	[85,86]
		Listeriolysin O (LLO)	Finestin, RD-1	[87,88]
	Decoy Capture	Hla, PLY, and potentially broad-spectrum	(Ch:Sm) liposomes, (Ch:Sm+Sm) liposomes, CAL02 (Combioxin SA)	[91]
		HLa, SLO, and potentially broad spectrum	Biomimetic RBC-coated nanoparticles	[95,96,97,98,99]
**Inhibition of Host Cell Receptors or Uptake Mechanisms**	Small Molecules, Drug Repurposing, Peptides and Antibodies	*S. aureus* α-toxin (Hla)	GI254023X (ADAM10 inhibitor)	[101,102,103]
		Intermediolysin (ILY)	ILY peptide, rILYd4	[108,109]
		*S. aureus* LeukED	Maraviroc (CCR5 agonist)	[104,105]
		Pneumolysin (PLY)	Anti-LeX/sLeX antibodies	[86]
		PLY, SLO, tetanolysin	Simvastatin	[113,114]
**Blockade of Pore Formation**	Small Molecules	*S. aureus* α-toxin (Hla)	β-cyclodextrins, isatin-Schiff base copper (II) complex	[122,123,124,125]
		*Clostridium perfringens* Etx	Specific quinoline and isoxazol compounds	[126]
**Increase Host Cell Resiliency Against PFT**	Stimulate Membrane Repair Pathways	Pneumolysin (PLY)	AP301 TNFα-derived TIP peptide, JI-34 GHRH agonist	[134,135]
		*S. aureus* α-toxin (Hla)	IFN-α (increase in lipid metabolism)	[133]
		*S. aureus* α-toxin (Hla)?	HIF-1 stabilizing PHD inhibitor, AKB4923	[137,140]
**Active Immunization**	Inactivated PFT (Toxoid) Vaccines	Pneumolysin (PLY)	Toxoid dPly, Δ6 PLY, peptide–L460D “pneumolysoid”	[153,154,155,156,157,158,159,160]
		*S. aureus* α-toxin (Hla)	Hla_H35L_, AT62, chimeric bivalent IsdB/Hla	[52,53] [161,162,163]
		Hla, possibly broad-spectrum	RBC “nanotoxoids” with absorbed PFTs	[171,172]
		*S. aureus* LukS-PV, LukF-PV	LukS-Mut9/LukF-Mut1	[64]
		Streptolysin O (SLO)	Inactivated W535A toxoid	[169,170]
		*Clostridium perfringens* Etx	Y30A-Y196A toxoid	[72,168]
		Listeriolysin O (LLO)	LLO W492A, LLO W491-492A toxoid	[164,165,166]
